# Multi-Echo Quantitative Susceptibility Mapping for Strategically Acquired Gradient Echo (STAGE) Imaging

**DOI:** 10.3389/fnins.2020.581474

**Published:** 2020-10-23

**Authors:** Sara Gharabaghi, Saifeng Liu, Ying Wang, Yongsheng Chen, Sagar Buch, Mojtaba Jokar, Thomas Wischgoll, Nasser H. Kashou, Chunyan Zhang, Bo Wu, Jingliang Cheng, E. Mark Haacke

**Affiliations:** ^1^Department of Computer Science and Engineering, Wright State University, Dayton, OH, United States; ^2^Magnetic Resonance Innovations, Inc., Bingham Farms, MI, United States; ^3^The MRI Institute for Biomedical Research, Bingham Farms, MI, United States; ^4^Department of Radiology, Wayne State University, Detroit, MI, United States; ^5^Department of Neurology, Wayne State University, Detroit, MI, United States; ^6^Department of Biomedical, Industrial and Human Factors Engineering, Wright State University, Dayton, OH, United States; ^7^Department of MRI, The First Affiliated Hospital of Zhengzhou University, Zhengzhou, Henan, China; ^8^Shanghai Zhu Yan Medical Technology Ltd., Shanghai, China; ^9^Department of Biomedical Engineering, Wayne State University, Detroit, MI, United States

**Keywords:** quantitative susceptibility mapping (QSM), constrained image reconstruction, gradient recalled echo (GRE) phase data, ill-posed inverse problem, strategically acquired gradient echo (STAGE) imaging

## Abstract

**Purpose:**

To develop a method to reconstruct quantitative susceptibility mapping (QSM) from multi-echo, multi-flip angle data collected using strategically acquired gradient echo (STAGE) imaging.

**Methods:**

The proposed QSM reconstruction algorithm, referred to as “structurally constrained Susceptibility Weighted Imaging and Mapping” scSWIM, performs an *ℓ*_*1*_ and *ℓ*_*2*_ regularization-based reconstruction in a single step. The unique contrast of the T1 weighted enhanced (T1WE) image derived from STAGE imaging was used to extract reliable geometry constraints to protect the basal ganglia from over-smoothing. The multi-echo multi-flip angle data were used for improving the contrast-to-noise ratio in QSM through a weighted averaging scheme. The measured susceptibility values from scSWIM for both simulated and *in vivo* data were compared to the: original susceptibility model (for simulated data only), the multi orientation COSMOS (for *in vivo* data only), truncated k-space division (TKD), iterative susceptibility weighted imaging and mapping (iSWIM), and morphology enabled dipole inversion (MEDI) algorithms. Goodness of fit was quantified by measuring the root mean squared error (RMSE) and structural similarity index (SSIM). Additionally, scSWIM was assessed in ten healthy subjects.

**Results:**

The unique contrast and tissue boundaries from T1WE and iSWIM enable the accurate definition of edges of high susceptibility regions. For the simulated brain model without the addition of microbleeds and calcium, the RMSE was best at 5.21ppb for scSWIM and 8.74ppb for MEDI thanks to the reduced streaking artifacts. However, by adding the microbleeds and calcium, MEDI’s performance dropped to 47.53ppb while scSWIM performance remained the same. The SSIM was highest for scSWIM (0.90) and then MEDI (0.80). The deviation from the expected susceptibility in deep gray matter structures for simulated data relative to the model (and for the *in vivo* data relative to COSMOS) as measured by the slope was lowest for scSWIM + 1%(−1%); MEDI + 2%(−11%) and then iSWIM −5%(−10%). Finally, scSWIM measurements in the basal ganglia of healthy subjects were in agreement with literature.

**Conclusion:**

This study shows that using a data fidelity term and structural constraints results in reduced noise and streaking artifacts while preserving structural details. Furthermore, the use of STAGE imaging with multi-echo and multi-flip data helps to improve the signal-to-noise ratio in QSM data and yields less artifacts.

## Introduction

Magnetic resonance imaging (MRI) offers many different contrast mechanisms. Today, it is possible to obtain magnetic susceptibility maps, $\chi \left(\vec{r}\right)$, of the human brain (and other parts of the body) that show the underlying tissue susceptibility distribution. Quantitative susceptibility mapping (QSM) data are reconstructed from phase information, which represents the magnetic field variations caused by the magnetization of an object in the presence of an external magnetic field (Haacke et al., 2015). The resulting susceptibility maps can be used to assess bleeding (Bilgic et al., 2012), calcium deposits (Deistung et al., 2013; Chen et al., 2014) and oxygen saturation (Haacke et al., 2010). The knowledge of the susceptibility source and the quantity of either iron or calcium can help improve the diagnosis of neurodegenerative diseases such as multiple sclerosis, Parkinson’s disease, stroke, Sturge-Weber syndrome and traumatic brain injury (Haacke et al., 2015) to name a few.

Extracting the susceptibility, χ, from Gradient Recalled Echo (GRE) phase data is an ill-posed problem because the dipole kernel has zeroes along a conical surface and, therefore, under-samples k-space (Haacke et al., 2015). Many studies have attempted to solve this problem. A fast and direct method to reconstruct χ is the Thresholded K-space Division (TKD) approach (Wharton et al., 2010) that uses a threshold to ignore the smaller values near the zeroes in the inversion process. However, the TKD reconstructed susceptibility map suffers from streaking artifacts and underestimates χ. An alternative approach referred to as iterative Susceptibility Weighted Imaging and mapping (iSWIM) has been used to fill in the missing parts of k-space to overcome these artifacts (Tang et al., 2013). This was accomplished by constraining the susceptibility values in regions with high susceptibility. However, the final images are still noisy in regions of uniform susceptibility. A better approach, in theory, but one that requires multiple scans, is the Calculation Of Susceptibility through Multiple Orientation Sampling (COSMOS) (Liu et al., 2009). This method utilizes the phase images from multiple orientations to stabilize the inversion process and remove the singularities by weighted linear least squares. This method is usually used as a gold standard in the evaluation of any QSM reconstruction method.

A number of other approaches use regularization techniques with different *a priori* information to reconstruct the susceptibility. Although, these methods are computationally more expensive than TKD approaches, the reconstruction times are still reasonable, and they are designed to smooth over regions that have homogeneous susceptibilities. For example, morphology enabled dipole inversion (MEDI) exploits the structural consistency between χ and the magnitude image in the form of an *ℓ*_*1*_-norm (Liu et al., 2012). However, this constraint can cause errors in regions where there are inconsistencies between the magnitude images and the susceptibility maps. Homogeneity Enabled Incremental Dipole Inversion (HEIDI) (Schweser et al., 2012) is another method that uses structural information from both magnitude and phase images to correct this issue. An alternative approach, structural feature based collaborative reconstruction (SFCR) (Bao et al., 2016), argues that the edge information from either magnitude or phase images does not reflect all the structural features in χ and the reconstructed image suffers from over-smoothed edges. The key steps in this approach are to include a structural feature-based *ℓ*_*1*_-norm constraint and a voxel fidelity-based *ℓ*_*2*_-norm constraint. This allows both edges and small objects to be recovered while still minimizing artifacts. Furthermore, most of these methods find the total field through a linear fitting of multi-echo phase data. However, the inclusion of long echo times can lead to blooming artifact, an increase in signal loss at the edges of the object and, potentially, an underestimation of χ.

Strategically acquired gradient echo imaging (STAGE) is a rapid multi-contrast multi-parametric imaging approach that employs two fully flow compensated double-echo GRE scans using low and high flip angles (FAs) relative to the Ernst angle of white matter. It provides not only a variety of qualitative images such as the T1weighted enhanced (T1WE) image, but also provides multiple quantitative information such as ${\text{R}}_{2}^{*}$, T1, and susceptibility maps (Chen et al., 2018b; Wang et al., 2018; Haacke et al., 2020). The T1WE image is generated from the combination of two GRE scans with low and high FAs (Chen et al., 2018b) where the radiofrequency (RF) transmit field variation is corrected (Wang et al., 2018). When compared with conventional T1W or T2^∗^W images, the T1WE images derived from STAGE have improved contrast between cortical gray matter and white matter, and between deep gray matter and white matter (Chen et al., 2018b). The improved contrast in the T1WE image benefits structural segmentation. STAGE has also become more broadly tested for a number of neurodegenerative diseases (Haacke et al., 2020). Therefore, in this study, we propose a “structurally constrained Susceptibility Weighted Imaging and Mapping” (scSWIM) method that reconstructs the susceptibility using multiple echo, multiple flip angle STAGE data. Similar to SFCR, scSWIM utilizes the structural information from both magnitude data and the susceptibility maps but in a single step. The scSWIM approach specifically uses the enhanced contrast available in STAGE imaging to define prior information about the edges of the white matter and gray matter. In this paper, we introduce scSWIM, evaluate it on simulated data and test it on *in vivo* brain data.

## Materials and Methods

### Calculating the Susceptibility From an L1 and L2 Norm Cost Function

Based on Maxwell’s equations, the relationship between the phase image φ(*r*) (obtained from a 3D GRE imaging approach) and susceptibility χ(*r*) in ppm (parts per million) can be written as (Haacke and Reichenbach, 2011):

(1) $$\varphi \left(r\right)=\gamma B_{0}\mathrm{TE}F^{-1}\left\{D\left(k\right)F\left\{\chi \left(r\right)\right\}\right\},$$

where *r*, *B*_*o*_ and *TE* are the voxel position vector in image domain, the main magnetic field strength (in T) and the echo time, respectively; γ = 2.675×10^8^*rad*/*s*/*T* is the gyromagnetic ratio; *F* and *F*^−1^ denote the Fourier and inverse Fourier transform operators, respectively; and *D*(*k*) is the Fourier transform of the unit dipole kernel at the position *k* = [*k*_x_, *k*_y_, *k*_z_] in k-space and is defined as:

(2) $$D\left(k\right)=\frac{k_{x}^{2}+k_{y}^{2}-2k_{z}^{2}}{3\left(k_{x}^{2}+k_{y}^{2}+k_{z}^{2}\right)}=\frac{1}{3}-\frac{k_{z}^{2}}{{\left|k\right|}^{2}}.$$

The objective function of scSWIM is similar to the S-step of SFCR (Bao et al., 2016) with changes in constraint definitions and is given as:

(3) $$\begin{array}{c} f\left(\chi \left(r\right)\right)=\frac{1}{2}{||W\left(r\right)\left(F^{-1}D\left(k\right)F\chi \left(r\right)-\delta B\left(r\right)\right)||}_{2}^{2}\\ \;+\lambda_{1}{||P\left(r\right)G\chi \left(r\right)||}_{1}+\frac{\lambda_{2}}{2}{||R\left(r\right)\chi \left(r\right)||}_{2}^{2}, \end{array}$$

and the final solution for the susceptibility is given by:

(4) $$\chi_{\mathrm{scSWIM}}\left(r\right)=\underset{\chi \left(r\right)}{argmin}f\left(\chi \left(r\right)\right),$$

where δ*B*(*r*) = φ(*r*)/(γ*B*_0_*TE*) and *W* in the data fidelity term is a weighting matrix proportional to the image magnitude that defines the reliability of the magnetic field shift for each voxel and *G* denotes the gradient operator.

In the S-step of the SFCR method, the edge matrix, *P*, is a binary mask that is derived from the initial susceptibility, $\hat{\chi }$ (where for convenience we have now dropped the dependence on r). This initial $\hat{\chi }$ (which is reconstructed from the first regularized minimization step of the SFCR, called the M-step) is based on an objective function that is similar to Eq. [3] but its constraints are based on the magnitude image. Also, *R* in the S-step of the SFCR method is a fidelity mask where voxels with high signal-to-noise ratio (SNR) are mapped to zero, low SNR to one and voxels corresponding to susceptibility artifact to two. However, the choice of *R*, *P* and the starting input are different for scSWIM as described below.

In scSWIM, we replaced the SFCR first regularized minimization (M-step) with iSWIM (Tang et al., 2013) since it has no smoothing, provides an initial susceptibility map with sharp vessels and the reconstruction times are short. Then, in the *l*_*1*_ regularization term of Eq. [3], we used the edge matrix, *P*, which is the binary mask that is derived from the product of the thresholded gradients of the T1WE image, *P*_T1WE_, and the initial susceptibility map,$P_{\hat{\chi }}$:

(5) $$P_{T1\mathrm{WE},i}=\left\{ \begin{array}{c} 0,\left|G_{i}\rho \right|\geq \mu_{1}\\ 1,\left|G_{i}\rho \right|<\mu_{1} \end{array}\right.\;\text{and}\;P_{\hat{\chi },i}=\left\{ \begin{array}{c} 0,\left|G_{i}\hat{\chi }\right|\geq \mu_{2}\\ 1,\left|G_{i}\hat{\chi }\right|<\mu_{2} \end{array}\right.,$$

where *G*_*i*_ denotes the gradient operator, *i* is an indicator to the x, y or z directions, and ρ denotes the T1WE image. Both μ_*1*_ and μ_*2*_ are threshold values chosen to be 2.5 times the noise level of the derivatives of ρ and $\hat{\chi }$, respectively, in order to maintain the edges of the gray/white matter, veins and other structures in the brain. Essentially, *P*_T1WE_ excludes the edges of the white matter and gray matter and $P_{\hat{\chi }}$ excludes the edges of the vessels and basal ganglia including the globus pallidus (GP), caudate nucleus (CN), putamen (PT), thalamus (THA), substantia nigra (SN), and red nucleus (RN) and *P* = *P*_*T*1*WE*_ × $P_{\hat{\chi }}$.

In the *l*_*2*_ regularization term, we have used a structural matrix *R* to protect voxels in the regions of high susceptibility, such as veins and basal ganglia structures, from being over-smoothed while still smoothing other regions. The matrix *R* is generated from the normalized T1WE image excluding the regions detected in the *R*_DGM_ (where DGM stands for “deep gray matter”) and $R_{\hat{\chi }}$ masks defined next. The *R*_DGM_ mask is calculated using an atlas-based segmentation method developed in-house (Wang et al., 2019). This method segments the deep gray matter structures from the high flip angle magnitude image (T1W), initial susceptibility map $\hat{\chi }$ and STAGE T1 weighted data and T1 maps. The $R_{\hat{\chi }}$ mask is generated from the method used in Tang et al. (2013) by applying a threshold to the homodyne filtered $\hat{\chi }$ map. Finally, the constants λ_1_ and λ_2_ are found using the L-curve approach (Hansen and O’Leary, 1992).

The single-echo scSWIM approach just described was then adopted to handle the multiple echo, multiple flip angle STAGE data. For this purpose, iSWIM was used as the initial input into scSWIM for the low flip angle, short echo data (FA_L_TE_1_). Then for the other three echoes (FA_H_TE_1_, FA_L_TE_2_, and FA_H_TE_2_), the reconstructed scSWIM from the previous echo was used as the initial guess for processing the scSWIM of the next echo. Finally, an averaged scSWIM was generated by an $R_{2}^{*}$-based weighted average of the individual echo scSWIMs (χ_i_):

(6) $$\chi =\sum_{i}w_{i}^{2}\chi_{i}/\sum_{i}w_{i}^{2},$$

where $w_{i}={\mathrm{TE}}_{i}e^{-{\mathrm{TE}}_{i}R_{2}^{*}}$ and $R_{2}^{*}$ is from the STAGE data and is created from averaging the $R_{2}^{*}$ maps from each of the flip angle images (Chen et al., 2018b; Wang et al., 2018):

(7) $$R_{2}^{*}=\frac{1}{{\mathrm{TE}}_{1}-{\mathrm{TE}}_{2}}\ln\left(\rho_{2}/\rho_{1}\right),$$

where ρ_*1*_ and ρ_*2*_ are the magnitudes of the first (*TE*_*1*_) and second (*TE*_*2*_) echoes, respectively.

**FIGURE 1:**
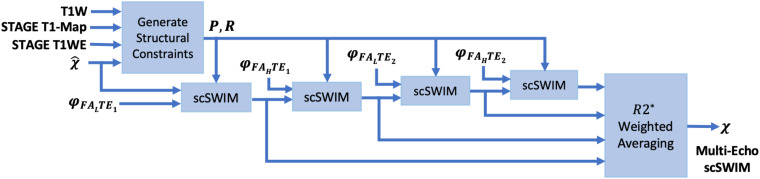
Block diagram of multi-echo, multi-flip angle scSWIM for STAGE imaging. Here, φ, $\hat{\chi }$ denote the phase and initial estimate of the susceptibility map from the multi-echo R2^∗^ weighted iSWIM, respectively. FA_*L*_ and FA_*H*_ denote the double-echo low and high flip angles scans of STAGE imaging, respectively.

This multi-echo approach has three advantages: first, each echo can be reviewed; second, the weighted scSWIM will have a better SNR; and third, loss of tissues associated with the use of a phase quality control map (especially at longer echoes) will be, to a large degree, replaced with the shorter echo scSWIM value. This weighting automatically ensures that wherever there is a measured susceptibility from one echo it will contribute to the final QSM result (while echoes with zeroes will not make a contribution). Figure 1 shows the block diagram of the proposed multi-echo, multi-flip angle scSWIM processing steps for STAGE.

### Simulated Data

The 3D isotropic susceptibility model developed in this laboratory (Buch et al., 2012) was used to test the algorithm. This model includes the general structures of the human brain such as gray matter (GM), white matter (WM), basal ganglia and midbrain structures [PT, GP, CN, THA, RN, SN, and crus cerebri (CC)], cerebrospinal fluid (CSF) and the major veins. The susceptibility values for these structures are summarized in the first row of Table 1.

**TABLE 1:** Susceptibility, T_1_ relaxation time, and relative proton density (ρ_0_) values for different structures in the simulated brain model.

	WM	GM	GP	PT	THA	CN	SN	RN	CC	V	VNT/CSF
χ (ppb)	0	20	180	90	10	60	160	130	−30	450	−14
T_1_ (ms)	837	1607	888	1140	1218	1226	1147	833	780	1932	4163
ρ_*0*_	0.73	0.80	0.72	0.82	0.79	0.82	0.79	0.80	0.79	0.85	1.00

*WM, white matter; GM, gray matter; GP, Globus Pallidus; PT, Putamen; THA, Thalamus; CN, Caudate Nucleus; SN, Substantia Nigra; RN, Red Nucleus; CC, Crus Cerebri; V, Veins; VNT, Ventricles; CSF, Cerebrospinal Fluid; and ppb, parts per billion.*

To test the performance of the reconstruction in the presence of cerebral microbleeds (CMB) or calcium deposits (CaD), two spherical objects with susceptibility values (radius) of 1000 ppb (5 mm) and 3000 ppb (3 mm), respectively, were added to the frontal white matter and two spherical objects with susceptibility values of −1000 ppb (5 mm) and −3000 ppb (3 mm) were added to the posterior white matter. Additionally, one spherical object with a radius of 3 mm with a susceptibility of −3000 ppb was added to the model to mimic the pineal gland (PG). The values for CMBs were taken from our experience in the field of traumatic brain injury and stroke where we usually see CMBs with susceptibilities as large as 1000 ppb but on occasion higher values up to 2000 ppb and 3000 ppb have been seen so we used both 1000 ppb and 3000 ppb to test the metal of the method. For the CaD, the values are around −3000 ppb but can range lower and slightly higher than this as the calcium is highly diamagnetic (Buch et al., 2015).

This final susceptibility model, χ_ideal_, was used to generate the magnitude and phase images using the STAGE imaging parameters: FA = 6^o^/24^o^, TE_1_ = 7.5/8.75ms, TE_2_ = 17.5/18.75ms, and TR = 25ms. The phase images were simulated from the forward model in Equations [1] and [2] at B_0_ = 3T. To create the magnitude images, first an R2^∗^ map was generated from χ_ideal_ using the relationship R2^∗^ = 20/s + 0.125χ (Ghassaban et al., 2019b) assuming R2^∗^ = 40/s for CMB, PG, and CaD objects. Then, the magnitude image was calculated using the Ernst equation (Brown et al., 2014). The proton density and T_1_ relaxation times for different brain structures are summarized in Table 1 while they are assumed to be zero for CMB, PG, and CaD objects. These values were adopted from the literature (Lee et al., 2006; Brown et al., 2014) or manually measured from the *in vivo* STAGE PD-map and T1-map.

Finally, Gaussian noise was added to the complex signal to produce an SNR of 10:1. The reconstructed susceptibility map using the proposed method was compared with the TKD, iSWIM, and MEDI methods. The original simulated susceptibility model (χ_ideal_) was used as the gold standard to measure the performance of each method using RMSE and SSIM as measures of goodness of fit (Wang et al., 2004) where SSIM = 1 corresponds to the perfect structural similarity while SSIM = 0 indicates no similarity between the two images.

### *In vivo* Data

The proposed scSWIM method was tested on two sets of *in vivo* datasets that are discussed below. All subjects involved in this study signed a consent form to be part of this IRB approved research.

#### Single Test Case Including COSMOS

The *in vivo* MRI data for this single test case was acquired from a 29-year old male volunteer on a 3T Siemens scanner (Siemens Healthcare, Erlangen, Germany) at Wayne State University. The imaging parameters were: 6° and 24° for the low and high flip angle scans with TR = 25 ms, TE_1_ = 6.5/7.5 ms, TE_2_ = 17.5/18.5 ms, bandwidth: 277 Hz/pixel, and GRAPPA = 2. The matrix size, voxel resolution, and FOV were 384 × 288 × 104, 0.67 × 0.67 × 1.33 mm^3^, and 256 × 192 × 139mm^3^, respectively. The total scan time for the high-resolution STAGE is about 10 min. For the purpose of generating COSMOS, two additional orientations with the same imaging parameters were collected for this subject. The reconstructed susceptibility map using the proposed scSWIM method was compared with those from the TKD, iSWIM and MEDI methods and compared to COSMOS as the reference image.

#### Evaluation on a Set of Healthy Human Subjects

Additionally, we tested scSWIM for ten healthy subjects acquired using a Siemens 3T Prisma scanner with lower resolution compared to the above-mentioned *in vivo* case. The imaging parameters were the same for the sample used above in the simulated data except the matrix size, voxel resolution, and FOV were 384 × 144 × 64, 0.67 × 1.33 × 2 mm^3^ (interpolated to 0.67 × 0.67 × 2 mm^3^) and 256 × 192 × 128 mm^3^, respectively, TE_1_ = 7.5/8.5 ms, and a bandwidth of 240 Hz/pixel. The total scan time for this resolution is about 5 min.

### Data Pre-processing

The entire processing pipeline was implemented in MATLAB (The Mathworks, Inc., Natick, MA, United States) on a workstation with Windows 10, Intel CPU i7-3770 with 4 cores and 16GB RAM. The phase image was first unwrapped using the bootstrapping (Chen et al., 2018a) and quality guided 3D phase unwrapping (Abdul-Rahman et al., 2007) methods in the simulated and *in vivo* data, respectively.

For the *in vivo* data, the induced background field from the air/tissue interfaces was removed from the unwrapped phase using the Sophisticated Harmonic Artifact Reduction for Phase data (SHARP) algorithm (Schweser et al., 2011) with a kernel size of 6 pixels. Next, the brain mask for the *in vivo* data was extracted from the magnitude images using BET (Brain Extraction Tool) (Smith, 2002). Finally, the resulting phase was zero-padded symmetrically in the spatial domain to a matrix size of 256 × 256 × 256 or 512 × 512 × 512 for simulated and *in vivo* datasets, respectively.

### Susceptibility Map Reconstruction

In Eq. [3], the parameters λ_*1*_ and λ_*2*_ were determined by plotting the measured residual errors of the data fidelity and the two regularization terms for each of the individual STAGE scans using the L-curve method (Hansen and O’Leary, 1992). In theory, λ_*1*_ controls the spatial smoothness and λ_*2*_ helps to preserve the high susceptibility regions and small objects such as vessels from being over-smoothed. As mentioned in section “Calculating the Susceptibility From an L1 and L2 Norm Cost Function,” an atlas-based segmentation method developed in-house (Wang et al., 2019) was used to generate the *R*_DGM_ mask. This method provided the labeled mask segmenting the right and left subcortical deep gray matter structures from the T1W, STAGE T1WE, T1 map, and $\hat{\chi }$. This labeled mask was carefully reviewed and if needed fine-tuned manually (this was done on 3 cases for the GP and SN structures which sometimes were smaller than what would have been drawn manually). If these regions had not been corrected, the algorithm would have smoothed that part of the GP not protected. Finally, the *R*_DGM_ mask is generated from binarizing the labeled mask.

Several algorithms were chosen to compare with scSWIM, including TKD, iSWIM, and MEDI. In generating the MEDI results, a regularization parameter of 250 (350) was used for the simulated (*in vivo*) data. For TKD processing, a threshold of 0.1 was used and iSWIM was performed with 4 iterations. All of these parameters were adjusted to give the lowest RMSE. Additionally, COSMOS was used as the gold standard for the *in vivo* data. Multi orientation images for the COSMOS data were co-registered using ANTs (Avants et al., 2009, 2012). In the TKD, iSWIM, and scSWIM methods, the final multiple echo, multiple flip angle QSM data were generated using a multi-echo R2^∗^-based weighted averaging of the individual QSM images from each echo and each flip angle data. In MEDI, the final QSM was generated by averaging the reconstructed QSM images from the fitted phases in each of the multi-echo low and high flip angle scans.

### Quantitative Analysis for Susceptibility Map

For the quantitative analysis of the data, the susceptibility mean and standard deviation were found from the entire 3D structure of interest. In the simulated model, all the structures of interest were measured automatically (since we know the location of each structure). For the *in vivo* data, the susceptibility of the midbrain structures were also automatically measured since they have been determined in creating the *R*_DGM_ masks for the boundaries of these structures as described earlier. On the other hand, the susceptibility of the CSF, WM, and major veins [SSV and internal cerebral vein (ICV)] were measured manually by tracing the ROIs on the QSM data using SPIN (SpinTech, Inc., Bingham Farms, MI. United States). The manual tracing was performed in the axial view for CSF and WM, but veins were traced in the sagittal view for easier localization. A linear regression model was used to compare the measured susceptibility values from each reconstruction method with those from the susceptibility model and COSMOS to assess the accuracy of midbrain structures in the simulated and *in vivo* data, respectively.

## Results

### Simulated Data

**FIGURE 2:**
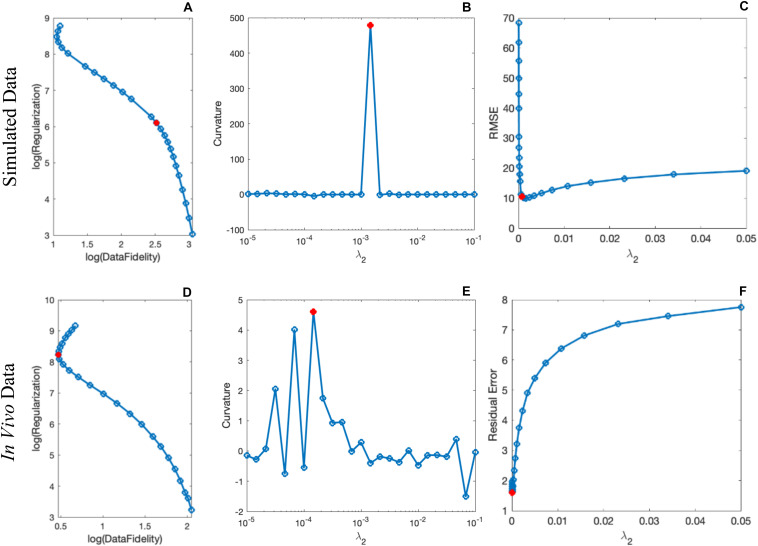
Determination of the scSWIM regularization parameter λ_2_ in the simulated **(A–C)** and *in vivo* **(D–F)** data for the higher flip angle, short echo (FA_H_TE_1_) scan using L-curve method. The curves in the first column show the log-log L-curve. The curvature and RMSE/residual error plot vs λ_2_ values are displayed in the third column. The optimal values (shown by the red circle) for the scSWIM at FA_H_TE_1_ scan were determined to be λ_2_ = 1.47×10^−3^ andλ_2_ = 1.47×10^−4^ for the simulated and *in vivo* data, respectively, where λ_*1*_ was set equal to 0.005λ_2_. This process is repeated for the other scans (FA_L_TE_1_, FA_L_TE_2_, and FA_H_TE_2_) and the optimal parameters were selected.

**FIGURE 3:**
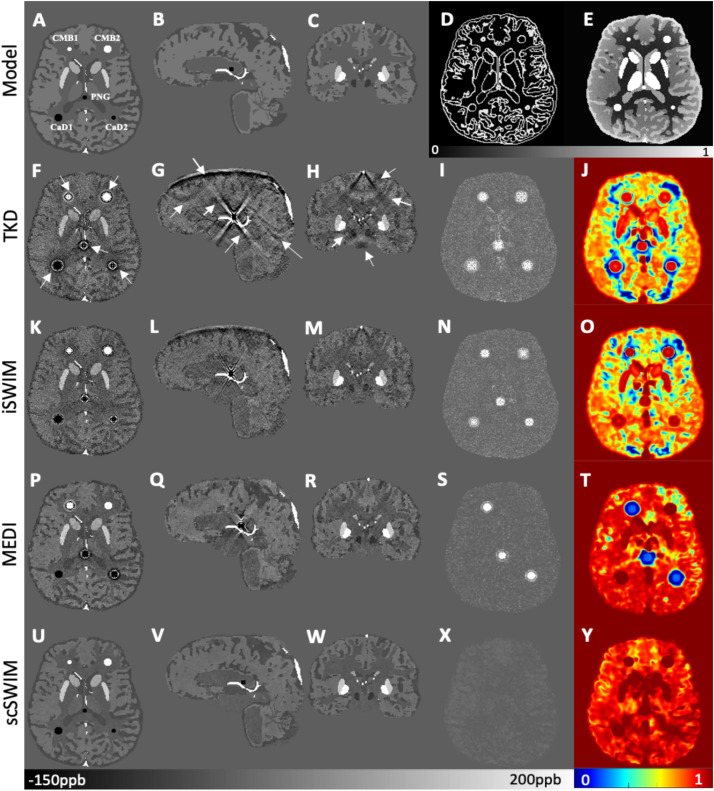
Depiction of multi-echo, multi-flip angle QSM images using different methods for the simulated data. This figure shows the orthogonal views of the susceptibility model **(A–C)**, and reconstructed QSM images from TKD **(F–H)**, iSWIM **(K–M)**, MEDI **(P–R)** and scSWIM **(U–W)** along with the scSWIM constraints *P*_ideal_ **(D)** and *R*_ideal_ **(E)**. The cerebral microbleeds (CMB), pineal gland (PG) and calcium deposits (CaD) are labeled on the model **(A)**. Streaking artifacts are indicated by the arrows. The last two columns show the corresponding susceptibility absolute error map **(I,N,S,X)** and structural similarity map **(J,O,T,Y)** for the different methods. In this simulated data, scSWIM provides better reconstruction with less artifacts, less error and higher similarity relative to the numerical model. Please note the complements of the *P* and *R* mask are shown in this figure **(D,E)** for better visualization.

By comparing the *P* and *R* masks for the simulated data (discussed in Section “Simulated Data”) and also the first and second regularization terms, and for the purpose of bringing the two terms to the same order, we set λ_1_ = 0.005λ_2_. This is further reviewed in the Discussion section. Based on this assumption and simulations in the human brain model, λ_2_ = {6.81,1.47,3.16,1.00}×10^−3^ provided the best results in terms of residual errors for the four different scans (FA_L_TE_1_, FA_H_TE_1_, FA_L_TE_2_, and FA_H_TE_2_), respectively (see Figures 2A–C for FA_H_TE_1_). A comparison of scSWIM with TKD, iSWIM, and MEDI along with their absolute errors and structural similarity maps relative to the simulation model are shown in Figure 3. In the simulated data (Figures 3A–C), we have used the exact known edge and structural matrices from χ_ideal_ to create *P*_ideal_ (Figure 3D) and *R*_ideal_ (Figure 3E). The TKD results (Figures 3F–J) show severe streaking artifacts while the iSWIM results have much less streaking (Figures 3K–O). MEDI does an excellent job (Figures 3P–T) as does scSWIM (Figures 3U–Y) in reproducing the model with minimal artifacts and noise. In both these last two reconstructions, the streaking artifact is highly reduced compared to both TKD and iSWIM and the images look much better in terms of SNR. However, MEDI does not resolve the streaking artifact from the CMB, pineal gland, or calcified objects with higher susceptibility values.

In the simulated data with (or without) CMBs, PG and CaDs, the RMSE for TKD, iSWIM, MEDI, and scSWIM were 32.91 (22.09), 24.61 (18.21), 47.53 (8.74) and 5.01 (5.21) ppb, respectively. Also, the SSIM index was measured as 0.52 (0.59), 0.62 (0.63), 0.80 (0.86) and 0.90 (0.91) for TKD, iSWIM, MEDI, and scSWIM, respectively, for these two conditions. Based on these results, scSWIM has the lowest error and the highest similarity to the model compared to the other methods. The measured susceptibility values in different structures are summarized in Table 2 showing that the measured susceptibilities in the midbrain structures for both MEDI and scSWIM are closer to the expected susceptibilities in the model while scSWIM has smaller standard deviations. The measured susceptibilities of the straight sinus vein, calcium deposition and CMB objects show that scSWIM provides the most accurate results in these structures as well.

**TABLE 2:** Measured susceptibility values (mean ± standard deviation) in ppb for different structures in the reconstructed QSM images using TKD, iSWIM, MEDI, and scSWIM methods for the simulated human dataset along with the reference values.

*Regions*	*TKD*	*iSWIM*	*MEDI*	*scSWIM*	*Model*
*CN-L*	44.17 ± 18.22	49.65 ± 15.46	54.55 ± 9.46	55.92 ± 2.41	60.00
*CN-R*	44.19 ± 18.67	49.19 ± 15.24	53.74 ± 9.94	55.42 ± 2.23	60.00
*GP-L*	152.17 ± 22.03	167.80 ± 18.41	172.06 ± 9.64	177.50 ± 2.74	180.00
*GP-R*	151.02 ± 20.65	166.44 ± 17.05	174.20 ± 9.93	175.94 ± 2.39	180.00
*PT-L*	74.77 ± 17.67	80.10 ± 14.94	84.66 ± 9.72	86.55 ± 2.42	90.00
*PT-R*	74.80 ± 17.78	78.84 ± 15.10	85.90 ± 9.28	85.51 ± 2.57	90.00
*THA-L*	3.31 ± 35.86	4.40 ± 24.97	3.44 ± 19.24	5.50 ± 2.35	10.00
*THA-R*	2.48 ± 30.50	3.36 ± 19.63	2.67 ± 14.17	5.11 ± 2.36	10.00
*WM*	−7.43 ± 14.35	−5.95 ± 12.33	−5.44 ± 7.31	−2.59 ± 1.66	0.00
*RN-L*	95.66 ± 36.19	129.41 ± 22.95	133.49 ± 10.63	131.22 ± 2.32	130.00
*RN-R*	95.96 ± 44.40	126.67 ± 22.72	135.58 ± 11.81	129.79 ± 2.51	130.00
*SN-L*	158.49 ± 32.41	151.66 ± 24.95	158.15 ± 11.43	159.16 ± 3.98	160.00
*SN-R*	139.56 ± 30.15	144.07 ± 22.01	154.88 ± 9.65	159.43 ± 4.71	160.00
*CC-L*	−30.85 ± 23.38	−28.67 ± 17.24	−36.24 ± 10.14	−31.28 ± 2.45	–30.00
*CC-R*	−32.08 ± 24.01	−26.41 ± 18.83	−37.56 ± 10.39	−30.50 ± 2.36	–30.00
*CSF*	−20.74 ± 19.21	−17.81 ± 13.12	−33.31 ± 11.31	−15.40 ± 2.25	–14.00
*SSV*	420.43 ± 61.28	447.76 ± 23.11	442.70 ± 12.95	450.83 ± 2.52	450.00
V	369.52 ± 85.28	408.74 ± 58.89	446.65 ± 48.29	446.90 ± 4.33	450.00
*CMB1*	3604.8 ± 709.84	2784.73 ± 772.89	958.42 ± 37.07	2992.54 ± 2.68	3000.00
*CMB2*	837.13 ± 97.45	922.31 ± 83.85	990.91 ± 12.36	995.58 ± 1.26	1000.00
*CaD1*	−855.56 ± 98.61	−970.63 ± 85.75	−995.38 ± 17.41	−1002.92 ± 1.42	–1000.00
*CaD2*	−3617.62 ± 715.04	−3914.88 ± 770.67	−1084.43 ± 8.72	−3002.70 ± 2.30	–3000.00
*PG*	−3605.53 ± 692.29	−3885.88 ± 760.06	−1053.80 ± 47.14	−2998.92 ± 1.97	–3000.00

*The susceptibilities for the left and right CN, GP, PT, THA, RN, SN, and CC were measured. CN, Caudate Nucleus; GP, Globus Pallidus; PT, Putamen; THA, Thalamus; WM, White Matter; RN, Red Nucleus; SN, Substantia Nigra; CC, Crus Cerebri; CSF, Cerebrospinal Fluid; SSV, Straight Sinus Vein; V, mean of all Veins; CMB, Cerebral Micro Bleed; CaD, Calcium Deposit; PG, Pineal Gland; L, Left; R, Right; and ppb, parts per billion unit.*

### *In vivo* Data

**FIGURE 4:**
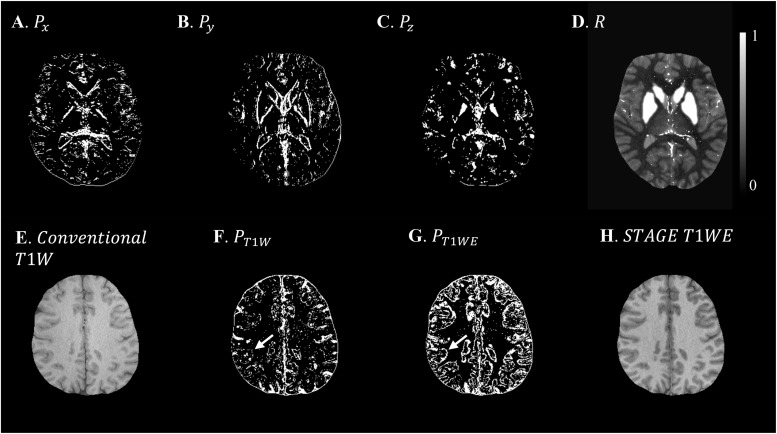
Illustration of scSWIM constraints and comparison of constraints extracted from conventional T1W and STAGE T1WE for the single high-resolution *in vivo* data. The first row shows the scSWIM structural constraints for the single high-resolution *in vivo* data: edge matrix, *P*, in the *x*, *y*, and *z* directions **(A–C)**, and the structural matrix, *R* **(D)**. The second row shows the advantage of extracting the constraints from STAGE versus conventional GRE data: conventional T1W **(G)**, STAGE T1WE **(H)**, and the extracted edges (product of three directions) from conventional T1W **(F)** and STAGE T1WE **(G)**. As seen, **(G)** provides more information about the white and gray matter edges (white arrow) and is less noisy than **(F)**. Please note the complement of the *P* and *R* mask is shown in this figure for better visualization.

Based on the L-curve analysis using the single high resolution human *in vivo* data (discussed in Section “Single Test Case Including COSMOS”) and by assuming λ_1_ = 0.005λ_2_ for the purpose of bringing two regularization terms to the same scale, λ_2_ = {1, 1.47, 1.00, 1.00}×10^−4^ provided the best results in terms of residual errors for FA_*L*_TE_1_, FA_*H*_TE_1_, FA_*L*_TE_2_, and FA_*H*_TE_2_, respectively (see Figures 2D–F). The structural terms used in the scSWIM cost function are illustrated in Figure 4. Specifically, Figures 4A–D show the edge and structural matrices *P* (includes *P*_x_, *P*_y_, and *P*_z_) and *R*. The binary matrix *P*, excludes the extracted edges from the enhanced T1-weighted and initial susceptibility while the binary mask *R* excludes the deep gray matter structures, vessels and other high susceptibility regions (the complement of *P* and *R* masks are shown in the figure for better visualization). Figures 4E–H show the conventional T1-weighted (Figure 4E) and T1WE (Figure 4H) from STAGE and their corresponding extracted edges (final P representation of extracted edges in three directions). It can be seen visually that the contrast between gray matter and white matter of the T1WE is higher than the conventional T1W image and its corresponding edge matrix, *P*_T1WE_, provides more information about the edge.

**FIGURE 5:**
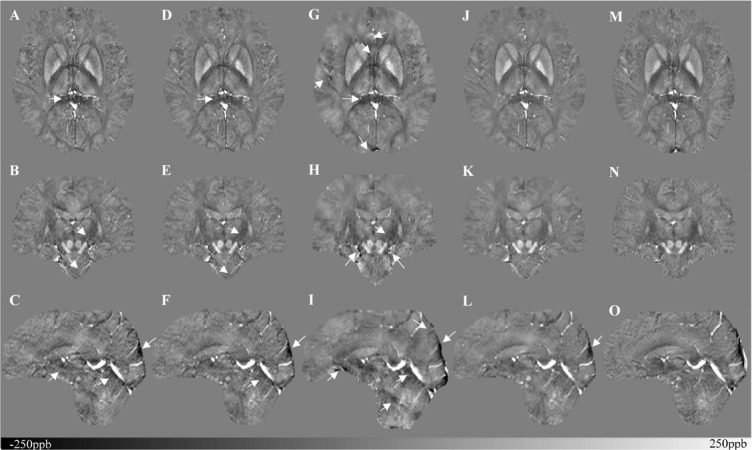
Depiction of multi-echo, multi-flip angle QSM images using different methods for the single high-resolution *in vivo* data. This figure shows three orthogonal views of the reconstructed multi-echo, multi-flip angle susceptibility maps from TKD **(A–C)**, iSWIM **(D–F)**, MEDI **(G–I)**, scSWIM **(J–L)**, and COSMOS **(M–O)** for the single high-resolution *in vivo* data. All of the images are displayed with the same window/level settings. White arrows show streaking artifacts. The SNR and image quality are best in the scSWIM images while the sharpness of the vessels and other brain structures are preserved.

Figure 5 shows three orthogonal views of the reconstructed multi-echo, multi-flip angle susceptibility images for this high-resolution human data set using the TKD (Figures 5A–C), iSWIM (Figures 5D–F), MEDI (Figures 5G–I), scSWIM (Figures 5J–L), and COSMOS (Figures 5M–O) methods. It can be seen in these images that scSWIM has less noise while the sharpness of the vessels and other brain structures are well-preserved. MEDI also provides a smooth reconstruction but in the regions that are close to the veins there are still some remaining artifacts. The measured susceptibility values in different structures are summarized in Table 3.

**TABLE 3:** Measured susceptibility values (mean ± standard deviation) in ppb for different structures in the reconstructed QSM images using TKD, iSWIM, MEDI, scSWIM, and COSMOS methods for the multi-echo, multi-flip angle in the single high-resolution *in vivo* data.

*Regions*	*TKD*	*iSWIM*	*MEDI*	*scSWIM*	*COSMOS*
*CN-L*	39.54 ± 29.34	39.13 ± 29.66	53.52 ± 33.58	50.63 ± 26.11	37.5 ± 34.1
*CN-R*	39.87 ± 29.36	38.11 ± 29.32	47.85 ± 28.98	51.95 ± 24.38	38.2 ± 32.5
*GP-L*	90.22 ± 48.18	98.32 ± 53.86	120.52 ± 59.17	125.28 ± 53.14	115.3 ± 66.3
*GP-R*	90.67 ± 42.59	98.92 ± 48.95	115.29 ± 43.19	123.89 ± 45.48	111.3 ± 55.0
*PT-L*	30.19 ± 32.36	29.58 ± 33.22	43.10 ± 35.50	50.24 ± 27.44	42.0 ± 32.0
*PT-R*	29.37 ± 32.43	29.31 ± 33.08	33.89 ± 34.56	47.45 ± 30.06	36.43 ± 32.95
*THA-L*	5.85 ± 32.00	2.82 ± 28.89	3.34 ± 39.32	6.72 ± 25.29	−1.89 ± 38.25
*THA-R*	7.47 ± 32.31	2.92 ± 30.28	7.16 ± 37.38	8.84 ± 25.41	−2.49 ± 38.77
*RN-L*	66.04 ± 28.71	66.63 ± 32.08	84.80 ± 35.88	99.46 ± 34.00	91.04 ± 48.14
*RN-R*	101.68 ± 35.38	113.07 ± 43.18	114.85 ± 39.88	120.85 ± 39.22	95.18 ± 53.17
*SN-L*	114.78 ± 67.60	129.69 ± 72.71	124.34 ± 78.56	140.86 ± 73.14	129.00 ± 81.30
*SN-R*	111.97 ± 58.00	124.66 ± 69.93	127.47 ± 69.16	147.67 ± 69.67	144.25 ± 79.86
*DN-L*	83.69 ± 36.22	86.79 ± 42.45	82.11 ± 36.57	93.37 ± 38.73	95.39 ± 44.07
*DN-R*	74.97 ± 35.69	82.21 ± 40.28	63.23 ± 39.63	92.17 ± 38.63	84.70 ± 47.20
*SSV*	424.62 ± 43.73	422.32 ± 43.65	395.39 ± 50.01	411.93 ± 42.46	404.95 ± 38.53
*ICV*	281.52 ± 59.40	298.13 ± 54.02	302.49 ± 54.77	326.07 ± 53.94	316.82 ± 67.50
*CSF*	16.96 ± 28.67	20.83 ± 26.19	26.65 ± 25.56	28.40 ± 22.33	18.54 ± 43.16
*WM*	9.67 ± 15.88	9.74 ± 15.48	13.12 ± 11.14	10.46 ± 9.71	1.06 ± 18.13

*The susceptibilities for the left and right CN, PT, GP, RN, and SN were measured. CN, Caudate Nucleus; GP, Globus Pallidus; PT, Putamen; THA, Thalamus; RN, Red Nucleus; SN, Substantia Nigra; DN, Dentate Nucleus; SSV, Straight Sinus Vein; ICV, Internal Cerebral Vein; CSF, Cerebrospinal Fluid; WM, White Matter; L, Left; R, Right; and ppb, parts per billion unit.*

**FIGURE 6:**
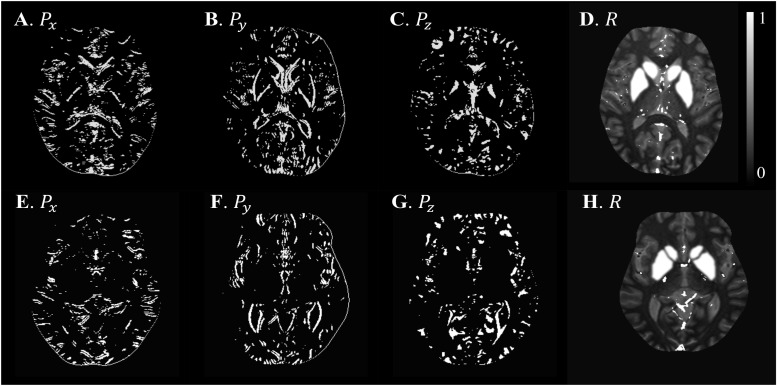
Illustration of scSWIM structural constraints for the two healthy subjects from the low-resolution dataset. The scSWIM structural constraints, edge matrix, *P*, in the *x*, *y*, and *z*, and structural matrix, *R*, are shown for the low- resolution *in vivo* data from a 62-year old healthy subject **(A–D)** and a 54-year old healthy subject **(E–H)**. Please note the complement of the *P* and *R* mask is shown in this figure for better visualization.

**FIGURE 7:**
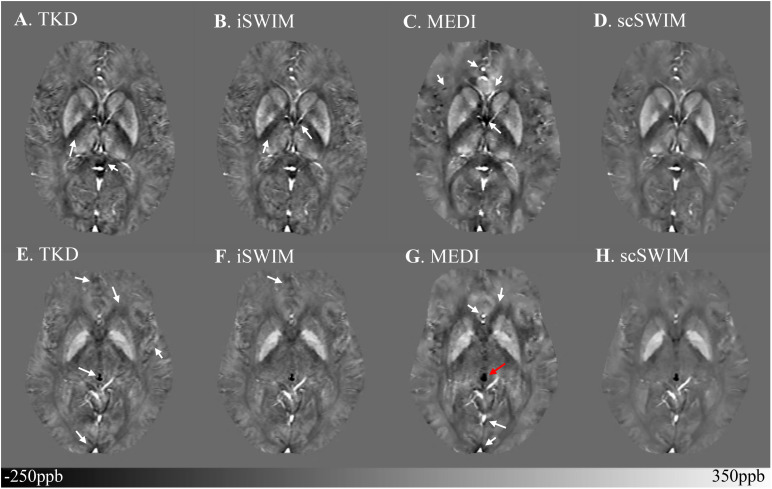
Depiction of multi-echo, multi-flip angle QSM images using different methods for the two healthy subjects from the low-resolution dataset. Multi-echo, multi-flip angle susceptibility maps from TKD **(A,E)**, iSWIM **(B,F)**, MEDI (λ = 350) **(C,G)**, and scSWIM **(D,H)** are shown for the two healthy subjects from Figure 6. The artifacts around the basal ganglia and larger veins in the TKD, iSWIM, and MEDI are shown by the white arrows. In the second row **(E–H)**, the pineal gland looks dilated in MEDI compared to other methods (red arrow).

The structural terms used in the scSWIM cost function for two selected healthy subjects with lower resolution data (discussed in Section “Evaluation on a Set of Healthy Human Subjects”) are illustrated in Figure 6. Here it can be seen that the edges are still well preserved with this *in vivo* STAGE approach. Figure 7 shows the reconstructed multi-echo, multi-flip angle susceptibility images using TKD (Figures 7A,E), iSWIM (Figures 7B,F), MEDI (Figures 7C,G) and scSWIM (Figures 7D,H) methods for two examples of this data. There are artifacts around the basal ganglia structures and larger veins in the TKD, iSWIM and MEDI (shown with white arrows). Furthermore, in the second slice (Figures 7E–H), the PG looks dilated in MEDI compared to the other methods (marked by a red arrow). Table 4 summarizes the averaged measured susceptibility values (mean ± standard deviation) in the reconstructed QSM images from the four different methods for the ten healthy subjects.

**TABLE 4:** Averaged susceptibility values (mean ± standard deviation) in ppb for midbrain structures in the reconstructed QSM images using TKD, iSWIM, MEDI, and scSWIM for ten healthy subjects from the low-resolution *in vivo* dataset from a Siemens 3T PRISMA scanner.

*Regions*	*TKD*	*iSWIM*	*MEDI*	*scSWIM*	*HC from Ghassaban et al. (2019a)*
*CN-L*	45.84 ± 9.05	44.22 ± 9.92	59.10 ± 13.33	52.77 ± 9.16	52.4 ± 7.6
*CN-R*	42.35 ± 9.16	40.76 ± 9.11	48.87 ± 13.62	50.93 ± 9.39	54.6 ± 6.6
*GP-L*	105.63 ± 16.15	118.45 ± 19.04	115.97 ± 20.53	129.13 ± 19.69	127.8 ± 7.8
*GP-R*	114.13 ± 21.33	126.85 ± 25.61	130.03 ± 24.41	136.12 ± 23.49	133.1 ± 10.1
*PT-L*	52.56 ± 17.32	54.82 ± 19	54.05 ± 19.39	68.71 ± 20.84	72.8 ± 7
*PT-R*	53.65 ± 22.91	56.19 ± 24.06	53.69 ± 22.51	67.50 ± 24.74	68.7 ± 6.4
*RN-L*	99.82 ± 21.34	109.65 ± 27.26	98.26 ± 17.49	111.51 ± 16.62	102.9 ± 12.9
*RN-R*	97.58 ± 25.33	106.64 ± 31.64	101.73 ± 26.64	108.91 ± 22.72	108.1 ± 13.0
*SN-L*	111.57 ± 14.12	123.87 ± 17.91	122.62 ± 11.74	128.05 ± 6.67	127.5 ± 10.8
*SN-R*	108.37 ± 18.54	120.58 ± 22.20	120.31 ± 24.55	123.83 ± 13.51	115.4 ± 11.6

*Also, the results from Ghassaban et al. (2019a) are summarized in the last column where the DGM structures are measured in both hemispheres in 24 healthy subjects from a GE 3T scanner. CN, Caudate Nucleus; GP, Globus Pallidus; PT, Putamen; RN, Red Nucleus; SN, Substantia Nigra; L, Left; R, Right; and ppb, parts per billion unit.*

**FIGURE 8:**
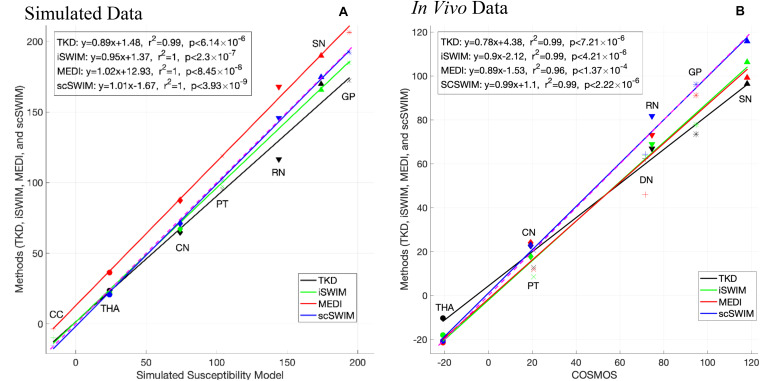
This figure shows the correlation of the susceptibilities of different basal ganglia structures (bilateral, that is the average of left and right) in the reference image with the ones in the reconstructed images using different methods in the simulated data **(A)** and *in vivo* data **(B)** [TKD (black), iSWIM (green), MEDI (red), and scSWIM (blue)]. All methods correlated well with iron content but scSWIM provided the best result relative to the correct absolute susceptibility. The dashed pink line corresponds to the line of identity between the individual reconstruction method and the reference susceptibility model and COSMOS for simulated and *in vivo* data, respectively.

Figure 8 shows the correlation between the zero-referenced estimated susceptibility for deep gray matter structures from different reconstruction methods with the actual susceptibility from the numerical model for the simulated data and reconstructed COSMOS for the *in vivo* data. The measured CSF susceptibility for each method is used to zero-reference the measurements. Among these methods, scSWIM (in blue color) has the closest values to the reference image in both datasets. The slope of scSWIM is 1.01(0.99) while TKD, iSWIM and MEDI are 0.89(0.78), 0.95(0.90), and 1.02(0.89) for simulated (and *in vivo*) data, respectively. The correlation coefficients in all methods are close to one and p-values to zero.

The current implementation of scSWIM for a single echo, converges in less than 3 and 5 iterations for the simulated and *in vivo* data, respectively. Each iteration consists of a minimization process that uses a preconditioned conjugate gradient solver. For the zero-padded *in vivo* data with a matrix size of 512×512×128, the total processing time for each single-echo scSWIM is currently 2∼5 minutes depending on the number of iterations using a Windows 10, Intel CPU i7-3770 with 4 cores and 16GB RAM.

## Discussion

The quantitative and qualitative analysis on both simulated data and human *in vivo* data showed that the reconstructed TKD suffers from streaking artifacts and underestimates the susceptibility values of deep gray matter and veins. The streaking artifact is reduced in iSWIM by using constraints from high susceptibility structures, but the final image is still noisy in the homogeneous regions. Thanks to the use of an *ℓ*_*1*_-norm regularization MEDI creates high SNR results. However, some streaking artifacts remain in regions where magnitude data is inconsistent with the susceptibility map. On the other hand, scSWIM uses both ℓ_1_ and ℓ_2_regularization terms to protect edges and structures while also allowing smoothing to increase SNR in regions without structure and it successfully reduces streaking artifacts leading to less noise and faithful estimates of the susceptibility. Furthermore, scSWIM outperforms other methods in reconstructing the susceptibility map in the presence of CMBs and CaDs with high susceptibilities. In simulated data, both microbleeds with susceptibilities of 1000 ppb and 3000 ppb and calcium objects with susceptibilities of −1000 ppb and −3000 ppb were reconstructed accurately using scSWIM compared to other methods. Also, in scSWIM, the standard deviation of the measured susceptibilities (Table 2) in all structures even in the CMB or CaD with the highest susceptibility values are much lower than other methods showing the strengths of this multi-echo approach. Although, MEDI provides a smooth QSM image under normal circumstances it appears to have trouble in reconstructing the data in the presence of high susceptibilities such as seen with the CMB and CaD in the simulated model and for the pineal gland in the *in vivo* data (which appeared dilated compared to that in scSWIM). This could be due to the fact that MEDI uses phase fitting across multiple echoes and high susceptibilities can cause both signal loss at the edge of the object and severe aliasing at longer echoes. Furthermore, in the *in vivo* data, one could observe slight streaking with MEDI around the large veins that could be due to the inconsistency between the magnitude and susceptibility data.

The *in vivo* results for scSWIM showed average susceptibilities for the ten healthy subjects very close to the reported values in the literature (Ghassaban et al., 2019a). Also, the measured susceptibilities in the reconstructed COSMOS (Table 3) were not as close to scSWIM and MEDI as one would have hoped because it can contained errors due to registration of the different orientation data and noise in the data. The registration error is higher and more noticeable in the regions near the surface of the brain. Luckily most of the regions of interest (the deep gray matter) in this paper are near the core of the brain where the registration error is smaller therefore this central region can still be used as a baseline to compare different methods.

### Structural Constraints in scSWIM

The cost function of scSWIM includes two regularization terms. The *ℓ*_*1*_-norm regularization term is based on a *P* mask to penalize the noisy non-edge pixels and the *ℓ*_*2*_-norm regularization term is based on the *R* mask that prevents smoothing in the excluded high susceptibility regions. If the pre-processing fails to extract the edges of a true structure, then the *P* mask will penalize and smooth them. On the other hand, if *R* fails to exclude a high susceptibility structure, the streaking artifacts from this structure will remain and its mean susceptibility will be reduced due to smoothing. This is because the *R* mask protects the structures of high susceptibility from being over smoothed by the *ℓ*_*1*_-norm regularization term. The overall performance of the cost function works well when the edges and structures are best defined.

### Optimal Parameter Selection for scSWIM

In the regularization-based approaches, there is always a trade-off between obtaining accurate susceptibility values, reducing streaking artifacts, and increasing SNR. In scSWIM, the λ_*1*_ parameter controls the spatial smoothness by applying the sparsity constraint on the gradient of the susceptibility map. The larger the λ_*1*_, the smoother the non-edge regions will be for both the background and basal ganglia (basically increasing the SNR). On the other hand, λ_*2*_ also controls smoothing the background but protects the objects defined by the *R* mask. Smaller λ_*2*_ reduces the effect of the regularization term and increases the effect of the data fidelity term and the streaking artifact will not be handled as well. On the other hand, larger λ_*2*_ will increase the effect of the regularization term and reduce the effect of the data fidelity term and will result in an over-smoothed image where the background such as WM and GM and smaller objects would be washed out.

Therefore, the challenging part of scSWIM is to find the optimal parameters to keep sharp edges, smooth where appropriate, and satisfy the data fidelity condition. However, finding optimal values for more than one parameter in regularization problems is still a difficult problem. With the admission of sub-optimality, we assumed that the ratio of λ_*1*_ and λ_*2*_ is fixed. For this purpose, we compared the *P* and *R* masks and also the first and second regularization terms and observed that λ_1_ = 0.005λ_2_ will bring the two terms to the same order. The final step was to determine the optimal value for λ_*2*_. This was accomplished using the L-curve approach that plots the residual data fidelity versus the regularization for different regularization parameters and selecting the value that results in the maximum curvature. For multi-echo, multi-flip angle scSWIM, the L-curves were analyzed for each individual scan separately and the optimal λ_*2*_ values selected accordingly.

### Multi-Echo, Multi-Flip Angle scSWIM

As mentioned before, STAGE imaging uses double-flip angle, double-echo GRE scans. The multi-echo, multi-flip angle scSWIM, or STAGE scSWIM is generated by an $R_{2}^{*}$-based weighted averaging of the individual echo scSWIM data sets. Besides having higher SNR in the STAGE scSWIM results, each individual scSWIM dataset can be reviewed separately if desired. It would be of interest to compare the QSM results with those from the R2^∗^ maps or even the T1maps given that iron can affect the T1 of tissue. Recently, there has been more interest in multi-contrast quantitative mapping in diseases such as Parkinson’s disease and dementia where a more systemic quantitative approach is being taken with 3D data. Iron has played a key role in these studies not just in the basal ganglia but also in the hippocampus, motor cortex and cortical gray matter in general.

More importantly, the final STAGE scSWIM will keep regions that have been removed by the phase quality control map at longer echo times. An alternate approach would be reconstructing QSM from the linear fit to the phase as done in MEDI. However, regions of high susceptibility phase aliasing can be severe and phase fitting may not be successful. Furthermore, severe loss of signal in and around the object (blooming artifacts) will occur for high susceptibilities that will result in a significantly under-estimated susceptibility. The use of shorter echo times and the weighting factors can favor the short echo data replacing the long echo data when the susceptibilities are very high as in the case of the CMBs and CaD as shown in the results section.

STAGE uses the conventional SWI with two flip angles and is effectively available at any site that can run 3D GRE imaging. It is a 5 min scan (2.5 min for each flip angle) that provides eight qualitative and seven quantitative clinically useful images such as T1maps, spin density maps, QSM, R2^∗^, B1 field corrections and etc. Although, the high resolution STAGE scan time may take longer (∼10 min), using a compressed sense factor of 3 to 4 the scan times can be brought back to a time frame of 5 to 7 min. The proposed scSWIM method achieved the best results when processing double-echo, double-flip angle STAGE data by using the derived T1WE images to extract reliable geometry constraints, but it can also be performed on a single-echo T1W SWI dataset.

## Conclusion

In this paper, we have proposed a constraint based QSM reconstruction algorithm scSWIM which uses STAGE and iSWIM inputs to reconstruct the susceptibility map from multiple flip angle, multiple echo data. The results show for both simulated and *in vivo* human brain data that streaking artifacts are suppressed, and SNR is increased. Further, the measured susceptibilities are accurate relative to the brain model used and scSWIM works well even for regions with high susceptibility such as microbleeds and calcifications.

## Data Availability Statement

The data analyzed in this study is subject to the following licenses/restrictions: The data is currently not available. The data is from a collaboration with a clinical site. Requests to access these datasets should be directed to Nmrimaging@aol.com.

## Ethics Statement

The studies involving human participants were reviewed and approved by Ethics Committee of Wayne State University and Ethics Committee of The First Affiliated Hospital of Zhengzhou University. The patients/participants provided their written informed consent to participate in this study.

## Author Contributions

SG and SL designed, executed, and implemented the research project under the supervision of EMH. SB, YC, YW, and MJ helped in designing the experiments. CZ and BW helped with data acquisition. JC was the senior supporter of the data acquisition. SG wrote the first draft of the manuscript. EMH, SL, and SB critically discussed the results and reviewed the manuscript. EMH, TW, YC, and NK provided feedback and helped shape the manuscript. All authors contributed to the article and approved the submitted version.

## Conflict of Interest

SG, MJ, and EMH are employees of the Magnetic Resonance Innovations Inc., Bingham Farms, MI, United States. YW and EMH are employees of The MRI Institute for Biomedical Research, Bingham Farms, MI, United States. The remaining authors declare that the research was conducted in the absence of any commercial or financial relationships that could be construed as a potential conflict of interest.
