# Supplementary material for: Multi-Echo Quantitative Susceptibility Mapping for Strategically Acquired Gradient Echo (STAGE) Imaging
Source: Front Neurosci. 2020 Oct 23;14:581474. doi: 10.3389/fnins.2020.581474 (PMC7645168; doi:10.3389/fnins.2020.581474)
Supplement: Supplementary file 1 [file Data_Sheet_1.DOCX]

Supplementary Material

**Image quality as a function of echo time versus a weighted summation of QSM data**

Signal-to-noise (SNR) in the QSM results are dependent on the magnitude in the original images and the echo time since phase increases linearly with echo time. The weighting factor used here assumed equal magnitude of the GM in both flip angles. However, one can just as easily use all the original magnitude images weighted by TE to perform the weighting. Implementing this gave nearly identical results to those presented here. Fixing the ratio actually avoids the inherent noise between the different flip angle data sets but is generally an approximation. In **Supplementary Figure 1**, we show the results from three slices to show the changes in image quality as the echo time increases for both flip angle data sets followed by the weighted average result. The second echo data sets have an SNR much higher than the first echo simply due to the longer echo times used and they show the gray matter/white matter contrast much better. The final averaged result looks similar to the second echo data sets but with slightly improved SNR. Table 1 below shows the susceptibility and standard deviation for the different structures and how the standard deviation decreases as echo time increases. Also, the weighted average ME scSWIM (see the last column of **Supplementary Table 1**) shows a tendency to give a $\sqrt{2}$ reduction in noise as expected although this will not always be the case if the noise is dominated by structural variability. In **Supplementary Figure 2**, we evaluate the Caudate using a profile approach to show the response across the boundary for the different QSM methods. This highlights where the contrast and contrast-to-noise in the QSM images are found to be comparable.

**Supplementary Table 1.** Measured susceptibility values (mean ± standard deviation) in ppb for different structures in the reconstructed scSWIM images from each of the short and long echoes of the small (FAL) and large (FAH) flip angles of STAGE scans compared to the combined multi-echo, multi-flip angle scSWIM in the single high-resolution *in vivo* data. These values are measured in the 2D region in a selected slice showing uniform susceptibility.

|  | *scSWIM_FALTE1_* | *scSWIM_FALTE2_* | *scSWIM_FAHTE1_* | *scSWIM_FAHTE2_* | *MEscSWIM* |
| --- | --- | --- | --- | --- | --- |
| *WM* | -15±34 | 5±19 | -10±18 | 0±8 | 1±8 |
| *CN* | 68±44 | 69±19 | 56±21 | 67±13 | 71±15 |
| *GP* | 152±56 | 139±29 | 160±36 | 145±20 | 143±15 |
| *PT* | 46±50 | 52±28 | 54±37 | 53±31 | 59±25 |
| *RN* | 120±51 | 118±28 | 126±31 | 145±20 | 143±15 |
| *SN* | 215±76 | 208±48 | 174±51 | 179±45 | 170±34 |
| *ICV* | 451±83 | 405±54 | 420±73 | 348±72 | 390±40 |
| *SSV* | 471±74 | 459±60 | 420±83 | 418±68 | 409±47 |

WM (White Matter), CN (Caudate Nucleus), GP (Globus Pallidus), PT (Putamen), RN (Red Nucleus), SN (Substantia Nigra), ICV (Internal Cerebral Vein), SSV (Straight Sinus Vein), CSF (Cerebrospinal Fluid), and ppb (parts per billion) unit.

**A**

**G**

**J**

**M**

| 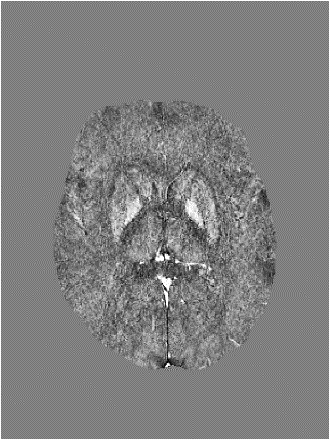 | 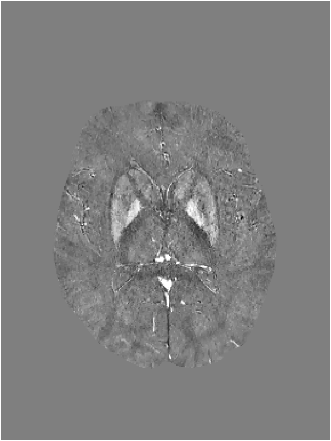 | 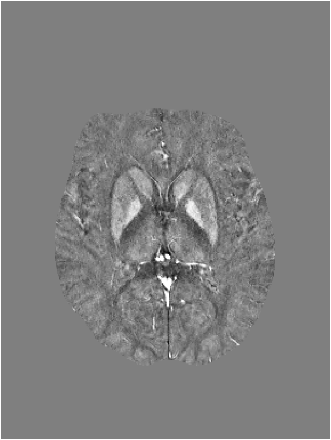 | 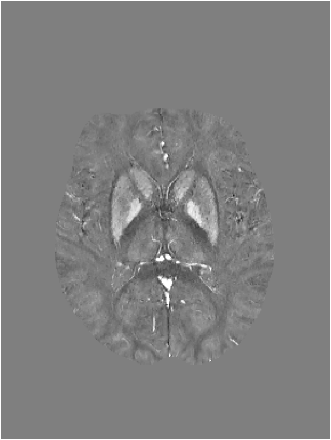 | 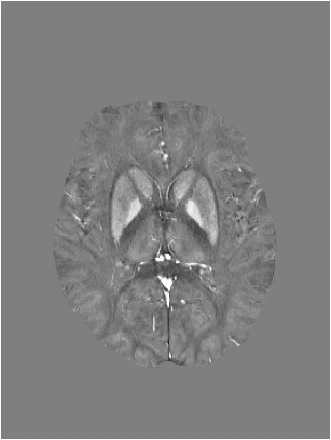 |
| --- | --- | --- | --- | --- |
| 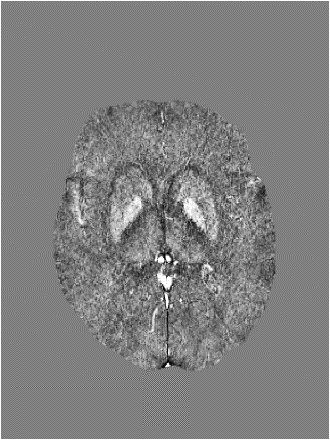  **B** | 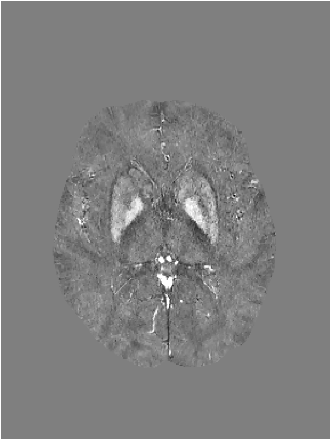  **D**  **E** | 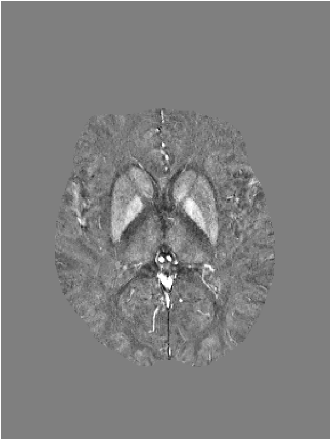  **H** | 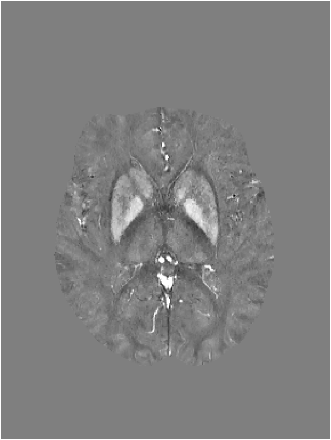  **K** | 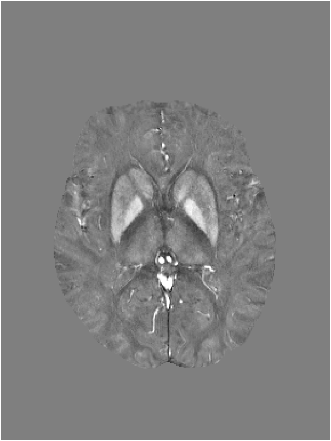  **N** |
| 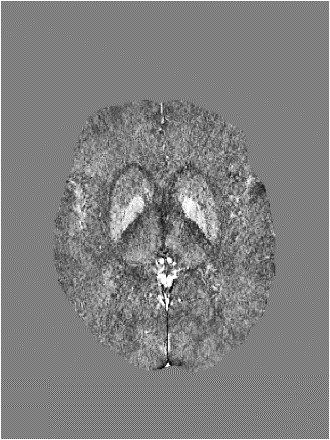  **C** | 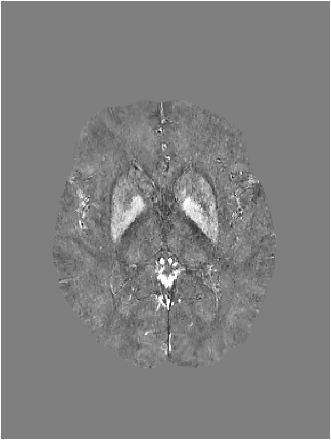  **F** | 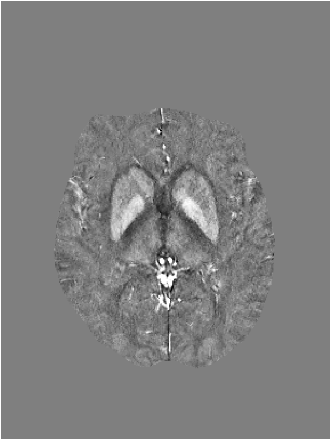  **I** | 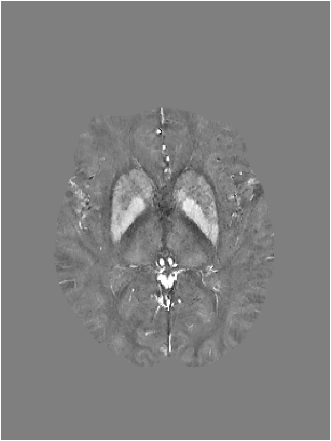  **L** | 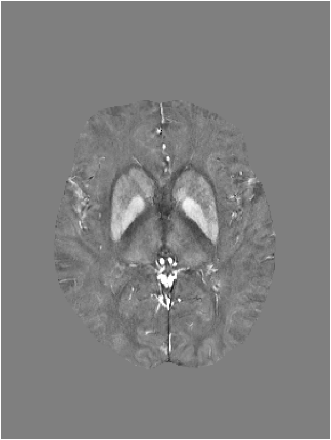  **O** |
| **Supplementary Figure 1.** Depiction of three consecutive slices of single-echo vs multi-echo, multi-flip angle scSWIM for the single high-resolution *in vivo* data. This figure shows three consecutive slices (57, 56, and 55) of the reconstructed scSWIM from single echo phase of FA_L_TE_1_ (**A-C**), FA_H_TE_1_ (**D-F**), FA_L_TE_2_ (**G-I**), and FA_H_TE_2_ (**J-L**) scans along with the multi-echo, multi-flip angle scSWIM (**M-O** ) for the single high-resolution *in vivo* data. All of the images are displayed with the same window/level settings. The SNR and image quality are best in the multi-echo, multi-flip scSWIM images while the sharpness of the vessels and other brain structures is preserved. | | | | |

| 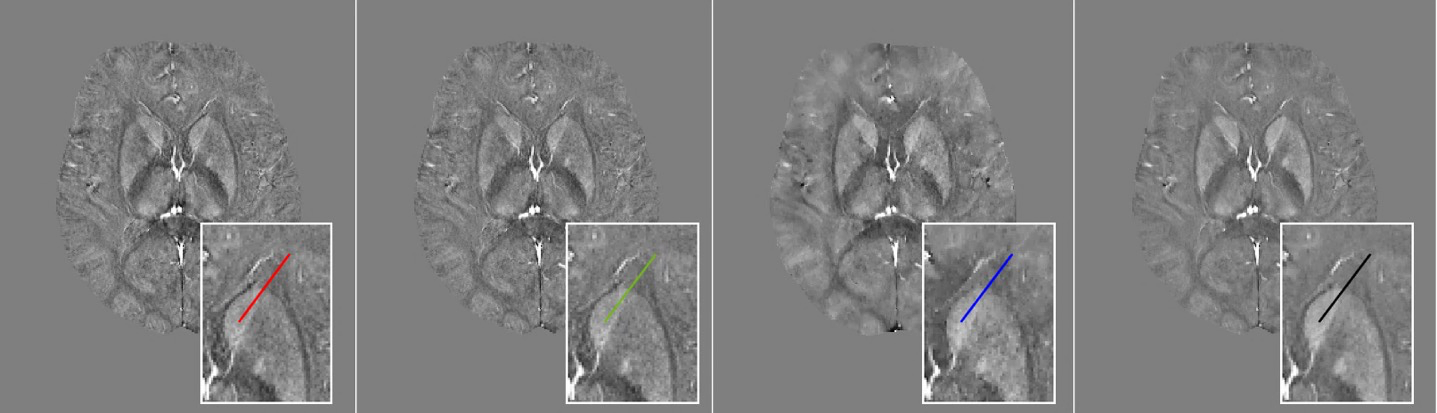  **D**  **C**  **B** | |
| --- | --- |
| 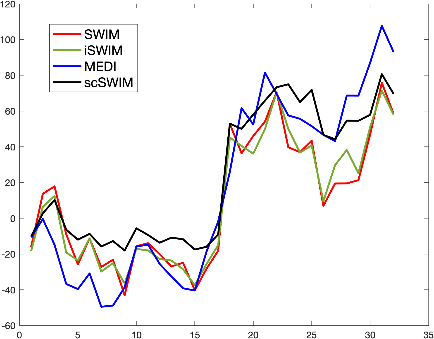  **E** | **Supplementary Figure 2.** Depiction of intensity profile of WM and GM in multi-echo, multi-flip angle QSMs using different methods for the single high-resolution *in vivo* data. This figure shows the reconstructed multi-echo, multi-flip angle susceptibility maps from TKD (**A**), iSWIM (**B**), MEDI (**C**), and scSWIM (**D**) for the single high-resolution *in vivo* data. The zoomed region including WM/GM are shown for each image and the intensity profiles along the depicted line in the zoomed images are shown in (**E**). As seen from the intensity profile scSWIM preserved the sharpness of the WM/GM boundary while it is less noisy than the other methods. |

**A**
